# An Immune Signature for Risk Stratification and Therapeutic Prediction in Helicobacter pylori-Infected Gastric Cancer

**DOI:** 10.3390/cancers14133276

**Published:** 2022-07-04

**Authors:** Haigang Geng, Zhongyi Dong, Linmeng Zhang, Chen Yang, Tingting Li, Yuxuan Lin, Shouyu Ke, Xiang Xia, Zizhen Zhang, Gang Zhao, Chunchao Zhu

**Affiliations:** 1 Department of Gastrointestinal Surgery, School of Medicine, Renji Hospital, Shanghai Jiao Tong University, Shanghai 200127, China; genghaigang@sjtu.edu.cn (H.G.); firgun123@sjtu.edu.cn (Z.D.); linyuxuan981222@163.com (Y.L.); keshouyu1019@sjtu.edu.cn (S.K.); xiaxiang@renji.com (X.X.); zhangzizhen@renji.com (Z.Z.); 2 State Key Laboratory of Oncogenes and Related Genes, Shanghai Cancer Institute, School of Medicine, Renji Hospital, Shanghai Jiao Tong University, Shanghai 200127, China; zlm_zlm@sjtu.edu.cn (L.Z.); chenyang_sim@sjtu.edu.cn (C.Y.); 3 State Key Laboratory of Genetic Engineering, Human Phenome Institute, Institute of Biostatistics, School of Life Sciences, Fudan University, Shanghai 200437, China; 20110700145@fudan.edu.cn

**Keywords:** Helicobacter pylori, gastric cancer, tumor immune microenvironment, prognosis, anti-PD-1 immunotherapy, dry lab

## Abstract

**Simple Summary:**

Helicobacter pylori (HP) infection is the greatest risk factor for gastric cancer (GC), and over half of the world’s population is colonized with HP. Up to now, many gene signatures are designed for predicting the prognosis of GC patients, while there are no signatures designed specifically for HP+ GC patients. Considering the tight association between HP infection and tumor immune microenvironment, we constructed an immune-related signature named IRSHG in this study, aiming to provide new insight into the treatment for HP+ GC patients. IRSHG was the first prognostic signature for HP+ GC patients, demonstrating high reliability and feasibility in predicting the prognosis. In addition, IRSHG could help investigate potential therapies and guide anti-PD-1 immunotherapy for HP+ GC patients, providing new insight for the personalized treatment of GC.

**Abstract:**

Helicobacter pylori (HP) infection is the greatest risk factor for gastric cancer (GC). Increasing evidence has clarified that tumor immune microenvironment (TIME) is closely related to the prognosis and therapeutic efficacy of HP-positive (HP+) GC patients. In this study, we aimed to construct a novel immune-related signature for predicting the prognosis and immunotherapy efficacy of HP+ GC patients. A total of 153 HP+ GC from three different cohorts were included in this study. An Immune-Related prognostic Signature for HP+ GC patients (IRSHG) was established using Univariate Cox regression, the LASSO algorithm, and Multivariate Cox regression. Univariate and Multivariate analyses proved IRSHG was an independent prognostic predictor for HP+ GC patients, and an IRSHG-integrated nomogram was established to quantitatively assessthe prognostic risk. The low-IRSHG group exhibited higher copy number load and distinct mutation profiles compared with the high-IRSHG group. In addition, the difference of hallmark pathways and immune cells infiltration between the two groups was investigated. Notably, tumor immune dysfunction and exclusion (TIDE) analysis indicated that the low-IRSHG group had a higher sensitivity to anti-PD-1 immunotherapy, which was validated by an external pabolizumab treatment cohort. Moreover, 98 chemotherapeutic drugs and corresponding potential biomarkers were identified for two groups, and several drugs with potential ability to reverse IRSHG score were identified using CMap analysis. Collectively, IRSHG may serve as a promising biomarker for survival outcome as well as immunotherapy efficacy. Furthermore, it can also help to prioritize potential therapeutics for HP+ GC patients, providing new insight for the personalized treatment of HP-infected GC.

## 1. Introduction:

Gastric cancer (GC) is the fifth most frequently diagnosed malignancy, causing the fourth-highest cancer-related death around the world [1]. For the treatment of GC, traditional treatments, including systemic chemotherapy, radiotherapy, surgery, and targeted therapy are the major treatment options, which have proven certain efficacy in prolonging the survival time of GC patients [2]. Despite the effectiveness of traditional treatments, recurrence and metastasis are still relatively common in advanced GC, leading to the lower 5-year survival rate (less than 20%) [3]. In recent decades, immunotherapy has emerged as a novel treatment strategy across a spectrum of malignancies, becoming a promising therapy for oncology treatment. Anti-programmed death-1 (PD-1) therapy, as the most representative immunotherapy, acts as an effective cancer therapy through inhibiting local immunosuppression in the tumor microenvironment (TME) and modulating T cell priming against tumor antigens in secondary lymphoid tissues [4]. According to the results of previous clinical trials (KEYNOTE—012, —059, —061, and —062) [5,6,7,8], a significant benefit was observed when the advanced GC patients were treated with pembrolizumab. Nevertheless, the clinical efficacy of anti-PD-1 therapy varies due to the tumor heterogeneity and the difference of TME [9,10,11]. Increasing evidence showed risk factors such as loss of neoantigens, cancer-derived exosomes, high tumor mutation burden, immune inhibitory molecules, and T cells exclusion might contribute to the drug resistance of anti-PD-1 therapy [12,13]. Systematical evaluation of the immune status based on immune-related genes (IRGs) and the precise prediction of prognosis or response to immunotherapies have become the key to the precision medicine for GC. Therefore, there is an urgent need for developing an IRGs-based signature to predict the prognosis and the immunotherapy efficacy for GC patients.

The microbiota is reported to be tightly associated with tumorigenesis and progression, which also serves as a key orchestrator of cancer therapy [14,15]. For the microbiota related to GC, Helicobacter pylori (HP), a kind of Gram-negative motile bacterium, has long been proved to be the most significant etiologic factor for GC [16,17,18]. Over half of the world’s population is colonized with HP, and the infection rate is even higher in some developing countries (>80%) [19,20]. Some HP strain-specific virulence factors, including VacA, CagA, and CagA pathogenicity island (Cag PAI) could induce the carcinogenesis of GC [21,22,23,24]. A recent randomized trial demonstrated that HP treatment for patients with early GC could effectively prevent metachronous cancer and reduce histologic changes [25]. Based on a meta-analysis, HP eradication is effective for the prevention and treatment of GC, which has proven to be a viable strategy to relieve the enormous disease burden of GC [26]. Furthermore, HP-positive (HP+) persons who had a family history of GC in first-degree relatives may show greater clinical benefit for the HP eradication treatment [16]. However, the eradication of HP cannot reduce the risk of GC to zero [27].

**Figure 1:**
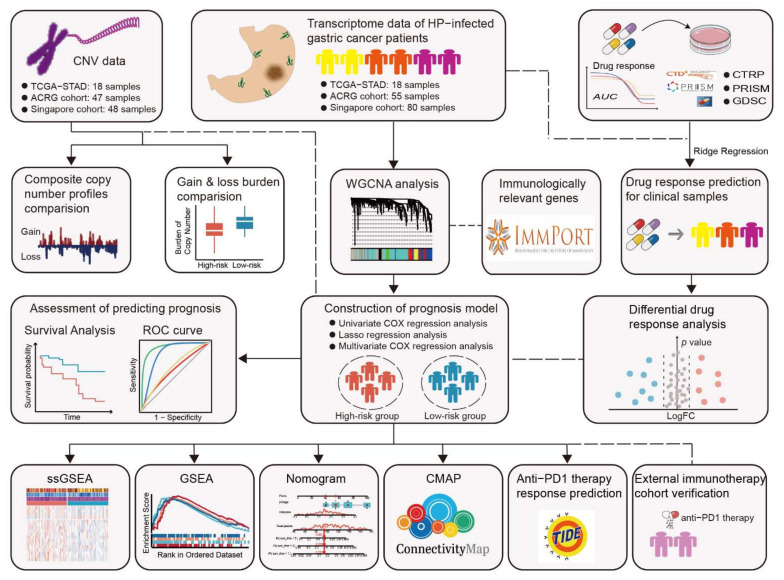
Flowchart for this work.

In the past decade, numerous molecular prognostic signatures have been proposed for GC, which helps to predict clinical outcomes and select therapy options for GC patients [28,29,30,31]. However, there were no signatures designed specifically for HP+ GC patients. Given the significant clinical and molecular differences between HP+ patients and HP-negative (HP−) patients, it is necessary to construct reliable signatures specific to different patient groups. Actually, the association between HP, tumor immune microenvironment (TIME), and malignancy has long been recognized in GC [32]. In this study, we firstly propose the Immune-Related prognostic Signature for HP+ GC patients (IRSHG) based on the transcriptome data, following a series of analyses to verify the reliability and effectiveness of IRSHG. In addition, we find that IRSHG could reflect the mutation characteristics and predict the sensitivity of anti-PD-1 immunotherapy for HP+ GC patients. The workflow for this study is shown in Figure 1.

## 2. Method

### 2.1. Transcriptome Data Sources and Preprocessing

Transcriptome data of patients with GC and corresponding clinical information were obtained from the Cancer Genome Atlas (TCGA, http://cancergenome.nih.gov/, accessed on 3 February 2022) and Gene Expression Omnibus (GEO, http://www.ncbi.nlm.nih.gov/geo/, accessed on 3 February 2022). After filtering out patients without HP infection status records, a total of 431 patients (including 155 HP+ GC patients and 276 HP—GC patients) were included in this study. Furthermore, two patients in HP+ group were excluded because of the short survival time and incomplete clinical information. A total of 153 HP+ GC patients, including 18 patients from TCGA-STAD [33], 55 patients from ACRG cohort [34], and 80 samples from Singapore cohort [35], were used for the subsequent analysis. The anti—PD—1 therapy cohort for gastric cancer (PRJEB25780) was obtained from ENA database (European Nucleotide Archive, https://www.ebi.ac.uk/, accessed on 3 February 2022) [36].

For RNA-sequencing cohorts, including TCGA-STAD and PRJEB25780 cohort, raw counts were transformed into transcripts per kilobase million (TPM) values for subsequent analysis. Raw expression data of microarray cohorts, including ACRG cohort and Singapore cohort, were processed with standard procedure and normalized with robust multi-array average (RMA) method located in the *affy* R package [37,38]. Transcriptome data from TCGA-STAD, ACRG cohort, and Singapore cohort were merged into one metadata set, and the batch effects were removed using the combat function located in the *sva* R package [39].

### 2.2. Weighted Gene Co-Expression Network Analysis (WGCNA)

A gene list containing 2483 immunologically relevant genes (IRGs) was extracted from the IMMPORT database. After taking the intersection of IRGs and the genes expressed in the metaset of HP+ GC patients, a gene expression matrix of 1183 unique IRGs and 153 HP+ GC patients was obtained, which was used to construct co-expression modules with *WGCNA* R package [40]. The best β value (soft thresholding power) was determined when the scale independence reached 0.9. Then, the genetic modules, which contained at least 50 IRGs, were identified for constructing the Pearson’s correlation matrix of the module-trait relationship between IRGs and immune traits (including immune score and ESTIMATE score). The modules with the high correlation to both immune score and ESTIMATE score were defined as immune-related modules and selected for further study. In addition, Gene Ontology (GO) enrichment and Kyoto Encyclopedia of Genes and Genomes (KEGG) pathway analysis were conducted for the functional annotation of genes in the immune-related modules using the *clusterProfiler* R package [41].

### 2.3. Construction of Immune-Related Prognostic Signature

To construct IRSHG, we randomly divided 153 HP+ GC patients into training and validation sets in the ratio of 7 to 3. Univariate Cox regression analysis was conducted to identify IRGs that were related to the prognosis of HP+ GC patients in immune-related modules. Then, the LASSO Cox regression algorithm was performed using *glmnet* R package [42], followed by multivariate Cox regression analysis for the construction of IRSHG. The formula of risk score for IRSHG was calculated as follows:

IRSHG score = IRG1 × IRG1 expression + IRG2 × IRG2 expression + · ···· + IRGn × IRGn expression.

### 2.4. Predictive Power Evaluation of IRSHG

HP+ GC patients in the training set, validation set, and total set were divided into the high- and low-risk groups according to the median risk score of IRSHG. To access the predictive power of IRSHG, five kinds of analyses were performed and described as follows:

- Receiver operating characteristic (ROC) curves: ROC curves of 1-, 3-, and 5-year survival were analyzed with the *timeROC* R package [43], and a high area under the curve (AUC) value indicated a high predictive power.
- Survival analysis: Survival analysis was conducted using Kaplan–Meier survival curves, which were utilized to compare the difference between the high-risk group and the low-risk group with a log-rank test
- Risk score plot: The risk score plot was used to visualize the grouping of risk factors in the prognostic model constructed by numerical type, which demonstrated the risk score distribution, survival status, and the expression of genes that made up IRSHG.
- Principal component analysis (PCA) and t-distribution stochastic neighbour embedding (t-SNE): To access the separating capacity of IRSHG, PCA and t-SNE were used to test the distribution of HP+ GC patients in the high-risk group and low-risk group.
- ROC values comparison: The 1-, 3-, and 5-year ROC values of IRSHG were compared with four previously published immune-related signature for GC, including Mao signature [44], Dai signature [45], Qiu signature [46], and Huo signature [47].

### 2.5. Copy Number Variation (CNV) Sources and Preprocessing

Raw copy number variation data carried out on the Affymetrix Genome-Wide Human SNP 6.0 Arrays were obtained in the form of cel files from GSE62717, GSE31168, and TCGA. Affymetrix Power Tools software was used for background processing, quality control, and signal intensity data normalization. Based on the PennCNV-Affy Protocol (http://www.openbioinformatics.org/penncnv/penncnv_tutorial_affy_gw6.html, accessed on 13 February 2022), an original signal file generated from Affymetrix Power Tools software was processed to obtain the LRR file and BAF file. To segment DNA copy number data, the circular binary segmentation (CBS) algorithm was implemented with *DNAcopy* R package [48]. Using the GISTIC 2.0 (GenePattern module) [49,50], copy number segment data were analyzed to identify significant focal- and arm-level copy number alterations in HP+ GC patents. Specially, the copy number status of each gene (including deletion, loss, gain, and amplification) was identified by applying both low- and high-level thresholds to the gene copy levels of all the samples. The difference of copy number burden between the high- and low-risk groups was calculated based on the total number of genes with focal- and arm-level copy number alterations using *t*-test. In addition, several cancer-related genes that varied significantly between high- and low- risk groups were demonstrated in a heatmap.

### 2.6. ESTIMATE Algorithm and Evaluation of Immune Infiltration

The Estimation of STromal and Immune cells in MAlignant Tumors using Expression data (ESTIMATE) algorithm was applied to infer the fraction of stromal and immune cells of GC patients using *estimate* R package [51]. The result of ESTIMATE analysis included immune score (indicating immune cell infiltration level), stromal score (indicating the presence of stroma in tumor tissue), ESTIMATE score (Comprehensive score based on immune score and stromal score), and tumor purity (indicating the proportion of cancer cells in tumor tissue).

To analyze related immune pathways and immune cell infiltration, we collected 33 immune cell signatures from previous reported research [52,53], and Single sample gene set enrichment analysis (ssGESA) was performed to quantify the enrichment level of immune signatures for every sample via the *GSVA* R package [54]. Moreover, the correlation between different immune cell infiltrating level was calculated in the high-risk group and low-risk group using Pearson correlation test.

### 2.7. Nearest Template Prediction (NTP) Analyses

Previously published GC molecular classifications, including Tan’s classification (G-DIF and G-INT) [55], Cao’s classification (Type I, Type II, and Type III) [56], and Lei’s classification (invasive, proliferative, and metabolic) [35], were predicted using NTP analyses (Gene Pattern modules) based on the provided subclass specific gene signatures, aiming to analyze the correlation between IRSHG and these GC molecular classifications.

### 2.8. Gene Set Enrichment Analysis (GSEA)

Fold change (FC) of each gene between the high-risk group and the low-risk group was calculated with the *limma* R package [57]. Based on the gene sets (c2.cp.kegg.v7.5.1.symbols) obtained from Molecular Signatures Database (MSigDB, http://software.broadinstitute.org/gsea/downloads.jsp, accessed on 13 February 2022), GSEA was performed to identify the enriched gene sets in the high-risk group and the low-risk group using the *clusterProfiler* R package. Pathways with *p*-value less than 0.05 were considered to be significantly enriched.

### 2.9. Construction of Predictive Nomogram

To identify the independent prognostic indicators for HP+ GC patients, univariate and multivariate Cox proportional hazards regression models were conducted using *survival* R package, and clinical characteristic with *p* value less than 0.05 was considered to be significantly related to the survival of HP+ GC patients. Pathological stage and IRSHG were used to construct the predictive nomogram to quantitatively access the prognostic risk for HP+ GC patients, and the 1-, 3-, and 5-year calibration curves were drawn for the examination of the predictive capability. In addition, the decision curve analysis (DCA) for 1-, 3-, and 5-year were used to measure the net clinical benefits for IRSHG, pathological stage, and the predictive nomogram.

### 2.10. Immunotherapy Response Prediction

TIDE (http://tide.dfci.harvard.edu/, accessed on 15 March 2022), an online algorithm for predicting the clinical response of patients to immune checkpoint blockade therapy, was performed based on the transcriptome data [58]. IRSHG score of each patient in PRJEB25780 cohort was calculated based on the formula of IRSHG, and the median IRSHG score of PRJEB25780 cohort was regarded as the high- and low-risk grouping criteria. Notably, the difference of immune therapy response between the high-risk group and the low-risk group was compared using Chi-square test.

### 2.11. Chemotherapeutic Drug Sensitivity Prediction

Using the “calcPhenotype” function located in the *oncoPredict* R package, the ridge regression model was used to predict chemotherapeutic drug sensitivity for HP+ GC patients based on the gene expression data [59]. The Sanger’s Genomics of Drug Sensitivity in Cancer (GDSC) and Broad Institute’s Cancer Therapeutics Response Portal (CTRP) data that were prepackaged into the *oncoPredict* R package were used for the training datasets. For drug response prediction of PRISM, the gene expression profile of cell lines was extracted from Cancer Cell Line Encyclopedia project (CCLE, https://portals.broadinstitute.org/ccle/, accessed on 22 August 2021), and drug response data of human cancer cell lines were obtained from PRISM Repurposing dataset (19Q4, released December 2019, https://depmap.org/portal/prism/, accessed on 22 August 2021). In addition, the IDWAS approach located in the *oncoPredict* R package was applied to estimate drug–gene interactions and identify biomarkers of drug response with linear models, which could identify the association between imputed drug response values and CNV data of HP+ GC patients. The result of IDWAS was visualized by Cytoscape (Version 3.9.1) [60].

### 2.12. Connective Map Analysis

Connective Map (CMap) was a large-scale data resource containing the expression profiles of five cell lines under 1309 different drug treatments, which was widely used for the in silico-based therapeutic discovery [61]. Recently, Yang et al. proved that eXtreme Sum (XSum) was identified to be an optimal method for matching compound and disease signatures in the recent extension of Cmap called Library of Integrated Network-based Cellular Signatures (LINCS), demonstrating better drug retrieval performance than five other available methods [62]. In addition, this study also claimed that the best prediction performance could be obtained when the query signature size was 100. Considering the difference of scale between Cmap and LINCS, top 150 genes positively/negatively correlated to IRSHG were selected using Pearson correlation analysis, which were used for potential drugs prediction.

### 2.13. Statistical Analysis

All statistical analyses were carried out using R statistics software (version 4.1.2). Unless specified otherwise, comparisons between two groups were analyzed by the Wilcoxon test. *p* < 0.05 was considered to be statistically significant.

## 3. Results

### 3.1. A Close Relationship between HP Infection and Immune Infiltration in GC

**Figure 2:**
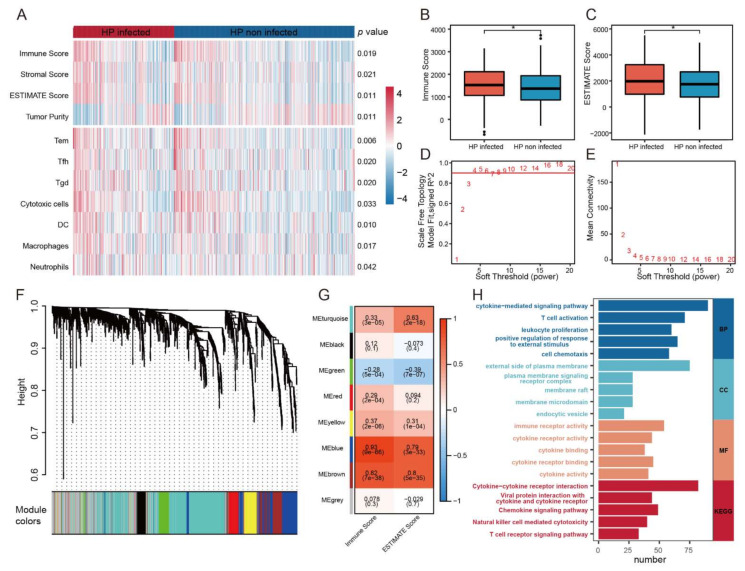
Comparison of immune infiltration between HP+ and HP− GC patients, and WGCNA analysis (**A**) Heatmap demonstrating the difference of immune cells infiltration score calculated by ssGSEA and the result of ESTIMATE algorism between HP+ GC patients and HP− GC patients. Boxplots illustrating the difference of immune score (**B**) and ESTIMATE score (**C**) between HP+ GC patients and HP− GC patients. * *p* < 0.05 (**D**,**E**) Scale-free fitting indices obtained by soft threshold analysis based on the topological network. (**F**) Clustering dendrogram of immune-related genes (IRGs) (**G**) Heatmap of the correlation between each module with the immune score and ESTIMATE score. (**H**) GO enrichment analysis and KEGG pathway analysis of IRGs in immune-related modules. (Abbreviations: BP: biological process; CC: cellular component; MF: molecular function; KEGG: Kyoto Encyclopedia of Genes and Genomes).

To explore the difference of immune status between HP+ GC patients and HP− GC patients, ESTIMATE algorithm and ssGSEA were carried out in 155 HP+ GC patients and 276 HP− GC patients. The result demonstrated that effective memory T cell (Tem), follicular helper T cells (Tfh), γδ T cell (Tgd), dendritic cells (DC), macrophages, and neutrophils were evaluated in HP+ GC patients (Figure 2A). Moreover, HP+ GC patients had higher immune scores and ESTIMATE scores than HP− GC patients, indicating the high-level immune infiltration of HP+ GC patients (Figure 2B,C). Therefore, it was of both clinical and scientific significance to construct an immune-related predictive signature specifically for HP+ GC patients. 

### 3.2. Construction of IRSHG via Co-Expression Network Analysis

**Figure 3:**
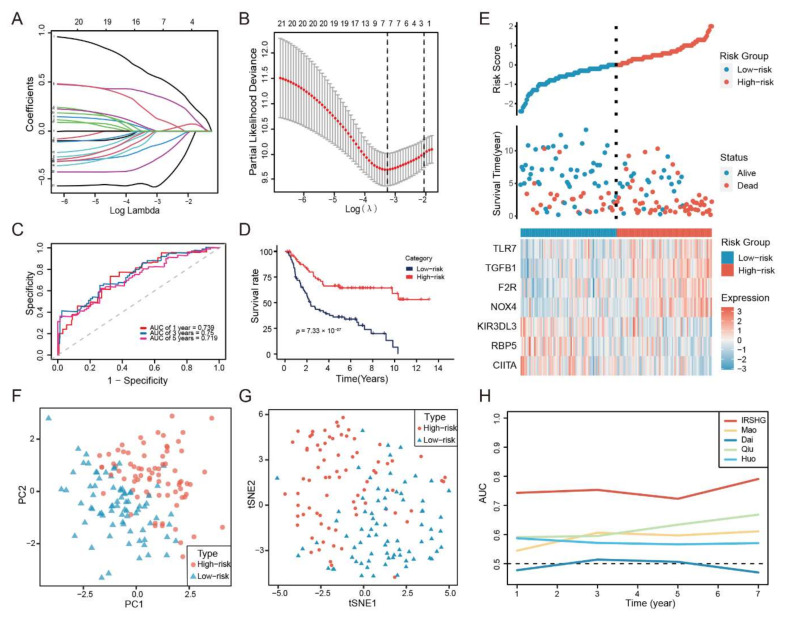
Construction of IRSHG and predictive power evaluation. (**A**) LASSO coefficients produced by LASSO regression analysis. (**B**) Lasso coefficient profiles of seven IRGs (**C**) ROC curve of 1-, 3-, and 5-year survival for the total set. (**D**) Kaplan–Meier survival curve for the total set. (**E**) Risk score plot showing the risk score distribution, survival status, and the expression of seven IRGs that made up IRSHG. PCA analysis (**F**) and t-SNE analysis (**G**) were performed on the high-risk group and the low-risk group based on the seven IRGs in IRSHG. (**H**) Time-dependent area under the ROC curve for the comparison of IRSHG with other four previously published prognostic signatures for GC.

WGCNA was performed on 153 HP+ GC patients to identify the immune-related gene modules based on the expression data matrix of IRGs. The optimal soft-thresholding power of 4 was chosen because it met the scale-free topology threshold of 0.92 (Figure 2D,E). The IRGs were clustered into eight modules using one-step network construction method, which was presented in a clustering dendrogram (Figure 2F). To identify immune-related modules, Pearson correlation analysis was used to analyze the correlation of these modules with immune score and ESTIMATE score. The module-trait association plot showed the blue module and brown module were highly correlated with immune score and ESTIMATE score (Pearson correlation coefficient > 0.7, *p* < 0.05), which were considered to be immune-related modules (Figure 2G). GO and KEGG enrichment analysis revealed that the 252 IRGs in immune-related modules mainly enriched in the biological process (BP) of cytokine-mediated signaling pathway, cellular component (CC) of external side of plasma membrane, molecular function (MF) of immune receptor activity, and KEGG pathway of cytokine-cytokine receptor interaction (Figure 2H, Table S1). Consequently, univariate Cox regression analysis was applied to analyze these IEGs, and 23 IRGs were proved to be survival-associated (Figure S1A). Then, LASSO Cox regression analyses and multivariate Cox regression were performed in the training set to construct IRSHG for predicting the prognosis of HP+ GC patients (Figure 3A,B and Figure S1B). The formula of IRSHG was as follows: IRSHG score = 0.706275 × TGFB1 expression + (−0.072151) × NOX4 expression + 0.462278 × F2R expression + 0.220028 × TLR7 expression + (−0.238008) × CIITA expression + (−0.466826) × RBP5 expression + (−0.795864) × KIR3DL3 expression.

### 3.3. IRSHG Has a Good Predictive Performance in Prognosis Prediction

HP+ GC patients in the training set, validation set, and total set were divided into the high-risk group and the low-risk group based on median IRSHG score. Five different analyses were performed to evaluate the predictive power of IRSHG. Firstly, ROC curve analysis of the total set showed good predictive capability of IRSHG (AUC = 0.739 for 1-year, 0.750 for 3-years, and 0.719 for 5-year survival) (Figure 3C). Secondly, for the total set, HP+ GC patients in the low-risk group were proved to have better prognoses than those in the high-risk group using Kaplan–Meier analysis (Figure 3D). Thirdly, risk score plot demonstrated the risk score distribution, survival status, and seven genes expression profile in the high- and low-risk groups (Figure 3E). These three analyses were also conducted on the HP+ GC patients in the training set and validation set, which further verified the predictive power of IRSHG (Figure S2A,B). Through PCA plot and t-SNE analysis, we found that HP+ GC patients in different risk groups could be well separated in two directions based on the seven genes of IRSHG (Figure 3F,G). Compared with four published prognostic signatures for GC patients, IRSHG had the highest AUC value in predicting the prognosis of HP+ GC patients (Figure 3H). Overall, the above results demonstrated the excellent predictive power of IRSHG, and the total set was used for subsequent study due to the limited sample size of HP+ GC patients.

### 3.4. CNV Analysis Reveals Different Mutation Profiles in the High- and Low-Risk Groups

**Figure 4:**
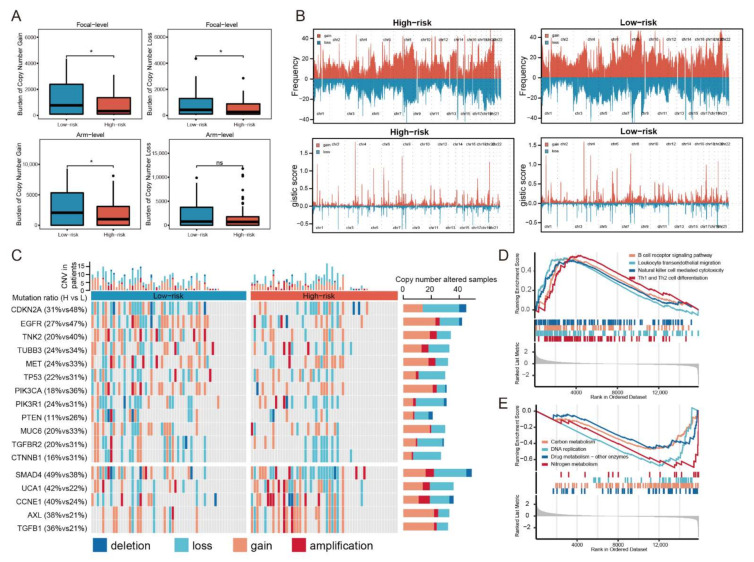
Copy number alteration landscapes and GSEA. (**A**) Comparison of the copy number load in focal-level and arm-level between the high-risk group and the low-risk group. * *p* < 0.05 (**B**) Distribution of the copy number gain and loss on chromosomes in the high- and low-risk groups. (**C**) Waterfall plot displaying the copy number mutation profile of the high- and low-risk groups. (**D**) Hallmark pathways enriched in the high-risk group. (**E**) Hallmark pathways enriched in the low-risk group.

Previous studies have reported that tumor microenvironment (TME) was closely associated with copy number alterations in tumor [63,64]. Thus, the difference of CNV between the high-risk group and the low-risk group was also analyzed. The CNV data of 55 patients in the high-risk group and 58 patients in the low-risk group was publicly available, which was used for subsequent analyses. For the copy number load, patients in the high-risk group showed a lower burden of copy number gain and loss than those in the low-risk group at the focal-level, and the burden of copy number gain at arm-level also fell in the high-risk group (Figure 4A). The distribution of the gistic-score and composite copy number alteration frequency across all chromosomes were compared between the high-risk group and the low-risk group, which was visualized in Figure 4B. For the high-risk group, significant amplifications demonstrated peaks in 3q26.1 and 20p13, while the frequently deleted genomic regions were 2q22.3, 8p11.22, and 16q12.2. Significant amplifications (3q26.1, 4q13.2, and 7q34) and deletions (1q44, 12p13.31, and 19q13.33) within chromosomal regions were identified in the low-risk group. In addition, mutation landscape of several driver genes was demonstrated across samples organized by IRSHG (Figure 4C). Compared with the high-risk group, the low-risk group had more mutations in several essential driver genes, such as *TP53*, *EGFR,* and *MET*, while mutations in *TGFB1*, *UCA1,* and *CCNE1* were more prevalent in the high-risk group. Notably, copy number alteration frequency of genes in PI3K signaling pathway (including *PIK3CA*, *PIK3R1,* and *PTEN*) was higher in the low-risk group. 

### 3.5. Hallmark Pathways Enriched in the High- and Low-Risk Groups

GSEA was conducted to investigate the cancer hallmark pathways associated with IRSHG. Several immune-related pathways, including B cell receptor signaling pathway, leukocyte transendothelial migration, natural killer cell mediated cytotoxicity, and Th1 and Th2 cell differentiation were upregulated in the high-risk group (Figure 4D, Table S2). By contrast, the low-risk group was enriched in some metabolic pathways, such as carbon metabolism, DNA replication, drug metabolism, and nitrogen metabolism (Figure 4E, Table S3).

### 3.6. The IRSHG-Integrated Nomogram Further Improves Prediction Ability for Prognosis

**Figure 5:**
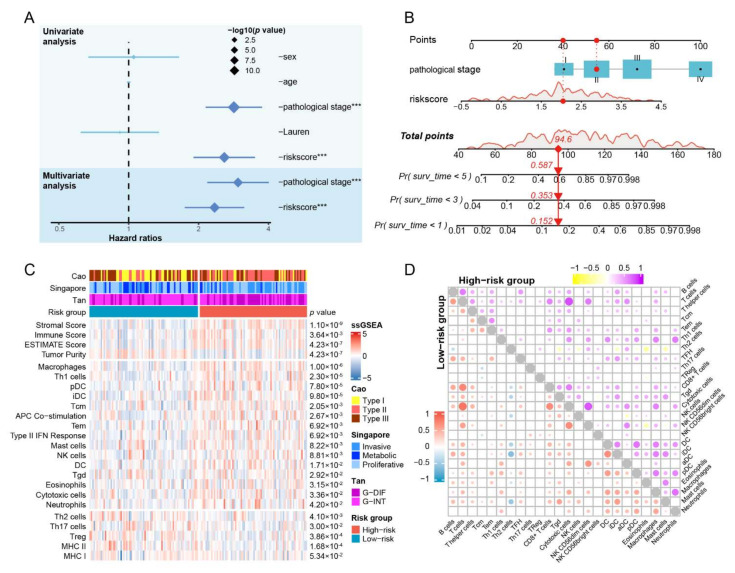
Establishment of nomogram and immune infiltration analysis. (**A**) Univariable analysis and multivariable analysis of clinical characteristics and IRSHG. *** *p* < 0.001 (**B**) Nomogram for predicting the probability of 1-, 3-, and 5-year overall survival in HP+ GC patients. (**C**) Heatmap illustrating the estimated scores of immune signatures calculated by ssGSEA and ESTIMATE algorism in the high- and low-risk groups. Previously reported transcriptome-based molecular classifications for GC were presented on the top of heatmap simultaneously. (**D**) Correlation of 24 immune cells in the high-risk group and the low-risk group, respectively.

Univariate and multivariate analyses were applied to integrate clinicopathological characteristics and IRSHG, aiming to identify independent prognostic indicators for HP+ GC patients. IRSHG and the clinical stage were determined as independent prognostic indicators for OS (*p* < 0.05), which were used for the construction of the prognostic nomogram (Figure 5A). To provide a quantitative instrument for predicting OS of HP+ GC patients, a prognostic nomogram built by IRSHG and the clinical stage was created (Figure 5B). The calibration plot of 1-, 3-, and 5-year OS demonstrated that the nomogram performed with moderate accuracy compared to an ideal model, indicating the excellent reliability and ideal consistency of the nomogram (Figure S3A–C). In addition, the DCA curves demonstrated that the IRSHG-integrated nomogram had a better net benefit compared with IRSHG and the pathological stage in predicting 1-year, 3-year, and 5-year OS of HP+ GC patients (Figure S3D–F).

### 3.7. Patients with High IRSHG Score Tend to Exhibit Higher Immune Abundance 

Multiple immune-related pathways were enriched in the high-risk group. Based on this observation, ESTIMATE algorithm and ssGSEA were conducted to explore the difference of immune cell infiltration between two groups (Figure 5C). Compared with the low-risk group, the high-risk group had higher immune score, ESTIMATE score, and most immune infiltration signatures, including macrophages, Th1 cells, plasmacytoid dendritic cells (pDC), interdigitating dendritic cells (iDC), central memory T cell (Tcm), APC Co-stimulation, Tem, Type II IFN response, mast cells, natural killer cell (NK), DC, Tgd, eosinophils, cytotoxic cells, and neutrophils. Meanwhile, Th2 cells, Th17 cells, Treg, and major histocompatibility complex class (MHC) II were significantly associated with the low-risk group. Notably, the low-risk group exhibited a tendency toward a higher MHC I score than the high-risk group, although this difference was not statistically significant (*p* = 0.053). Using the NTP analysis, the high-risk group was found to be related to previously reported transcriptome-based GC molecular subtypes, including Type II in Cao’s classification, invasive type in Lei’s classification, and G-DIF type in Tan’s classification. Meanwhile, the low-risk group was found to be closely associated with Type I in Cao’s classification, metabolic type in Lei’s classification, and G-INT type in Tan’s classification. The metabolic type was inconsistent with the metabolic pathways enriched in the low-risk group, indicating the reliability of the NTP analysis. To analyze the correlation of estimated absolute scores for each immune cell type by ssGSEA in the high- and low-risk groups, Pearson correlation analysis was conducted, and the result was showed in the double correlation heatmap (Figure 5D).

### 3.8. Patients with Low IRSHG Score Tend to Be Sensitive to Anti-PD1 Immunotherapy

**Figure 6:**
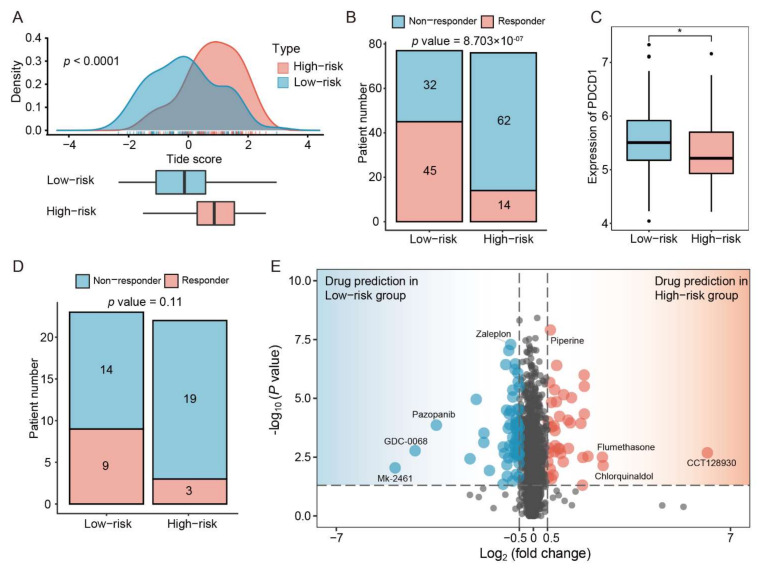
Immunotherapy efficacy accession and potential chemotherapy drugs prediction (**A**) The difference of the tumor immune dysfunction and exclusion (TIDE) score between the high- and low-risk groups. (**B**) Proportion of responders and non-responders in HP+ GC patients based on the result of TIDE algorithm. Statistical significance of difference was determined using Chi-square test. (**C**) The difference of PDCD1 expression between the high- and low-risk groups. * *p* < 0.05 (**D**) Proportion of responders (CR/PR) and non-responders (SD/PD) in patients treated with anti-PD-1 immunotherapy from the PRJEB25780 cohort. (**E**) Drug candidates with potential therapeutic effect for the low-risk group or the high-risk group.

To explore the relationship between IRSHG and immunotherapy, Tide analysis was performed to predict the immunotherapy response of HP+ GC patients in the high- and low-risk groups. The result showed that HP+ GC patients in the low-risk group had lower Tide score than those in the high-risk group, suggesting that they may benefit more from immunotherapy (Figure 6A). Meanwhile, more potential responders to anti-PD-1 inhibitors were observed in the low-risk group compared with the high-risk group using Chi-square test (Figure 6B). Therefore, we further compared the expression of *PDCD1* between the low-risk group and the high-risk group, and HP+ GC patients in the low-risk group were proved to have higher *PDCD1* expression (Figure 6C). Considering that HP+ GC patients in the low-risk group may be more sensitive to anti-PD-1 inhibitors than those in the high-risk group, an external anti-PD1 immunotherapy cohort PRJEB25780 was used to verify this prediction. Among 12 anti-PD-1 responders (including complete response and partial response) in the PRJEB25780 cohort, the IRSHG scores of nine responders were lower than the median IRSHG score of the PRJEB25780 cohort, although not statistically significant (*p* = 0.11) (Figure 6D). However, the PRJEB25780 cohort did not provide the HP infection status of each patient, thus this verification may only be used for reference.

### 3.9. Identification of Specific Chemotherapeutic Drugs Associated with IRSHG

After removing duplicate and invalid drugs (drugs with NA value in more than 20% of the samples), a total of 2005 drugs were obtained from three different pharmacogenomic databases (CTRP, GDSC and PRISM) for the chemotherapeutic drug sensitivity prediction. *OncoPredict*, an upgraded version of *pRRophetic* R package [65], was applied to predict the drug sensitivity for each patients using ridge regression model based on the gene expression matrix. The difference of imputed drug sensitivity was compared between the high- and low-risk groups. There were 98 drugs with statistical significance (|log2(fold change)| > 0.5, *p* < 0.05, 59 drugs for the low-risk group, and 39 drugs for the high-risk group) identified via this analysis (Figure 6E, Table S4). Interestingly, we found the response to these drugs might closely be associated with the CNV of HP+ GC patients. For example, patients in the low-risk had more *MET* and *EGFR* mutation than those in the high-risk group, which were more sensitive to Mk-2461 (c-MET inhibitor) and Pazopanib (VEGFR inhibitor) (Figure 6E). Therefore, with the IDWAS function in OncoPredict R package, drug response sensitivity data of 98 identified drugs and CNV data of HP+ GC patients were used to explore the potential biomarkers for the use of these drugs. Genes mutated with a frequency > 10% across all patients were included, which might render potential drugs ineffective or effective. Specific association between drugs and mutations was shown in a drug-mutation network (Figure S4, Table S5).

### 3.10. CMap Analysis Uncovers Drugs Which May Reverse IRSHG Score

Based on the basic concept called ‘signature reversion’ [66], the computational drug discovery was conducted to identify drugs with the ability to reverse IRSHG-associated gene expression pattern using CMap data (Figure S5A). Through Pearson correlation analysis, a total of 300 IRSHG-associated genes were identified (Figure S5B, Table S6). The result of CMap analysis uncovered several compounds of which gene expression patterns were oppositional to the IRSHG-specific expression patterns, and a lower CMap score indicated higher perturbation ability (Figure S5C, Table S7). In addition, some candidate drugs have been proven to be effective in GC with HP-infection. For example, AH-6809 (a kind of EP 2 antagonist, ranked third among all drugs) could inhibit HP-induced uPA and uPAR mRNA expressions, which suppressed the process of degradation of the extracellular matrix, tumor invasion, and metastasis of GC [67].

## 4. Discussion

A large part of the global cancer burden was attributed to carcinogenic infection, and HP was the most significant infectious agent worldwide [1,68]. Approximately 50% of the world population was infected with HP, which was considered to be the major cause of non-cardiac GC [69,70]. HP could enhance the inflammatory response and induce the occurrence of GC through a variety of ways. Canonical NF-κB signaling in gastric epithelial cells was activated through mechanisms depending on the HP’s Cag PAI-encoded type IV secretion system (T4SS), which could contribute to the DNA damage and oncogenic mutations [71,72]. The interaction between the activated Met and unphosphorylated CagA secreted by HP ensured the sustained activation of PI3K/Akt signaling, leading to the activation of β-catenin and NF-κB signaling [73]. Furthermore, the infection of HP could disturb the TME. Several pro-inflammatory cytokines including interleukin (IL)-1, IL-6, IL-8, and TNF-α were proved to be up-regulated in HP+ GC patients, leading to massive immune cell infiltration [74]. Nagase et al. demonstrated that the HP infection could upregulate the expression of TCR-inducible costimulatory receptor (ICOS) in pDC and Tregs, suggesting eradicating therapy for HP might serve as an indirect immune therapy for GC [75]. In addition, a previous study indicated that the PD-1 expression in peripheral blood and tumor infiltrating T (TIL) cells increased along with disease progression in HP+ GC patients [76]. Considering the tight association between HP infection and TIME, we constructed an immune-related signature named IRSHG in this study, aiming to provide new insight into the treatment for HP+ GC patients.

The establishment of IRSHG was based on seven IRGs (*TLR7*, *TGFB1*, *F2R*, *NOX4*, *KIR3DL3*, *RBP5,* and *CIITA*). Toll-like receptor 7 (TLR7) was reported to be involved in the recognition of HP-purified RNA, which could lead to the induction of pro-inflammatory cytokines and type I IFN production in a MyD88-dependent manner [77,78]. Choi et al. showed that HP+ GC patients were more likely to exhibit stronger immunostaining of TGF-β1 protein in noncancerous tissue compared with HP- GC patients [79]. Chronic inflammation caused by HP infection could contribute to the increased production of reactive oxygen species through phagocyte NADPH oxidase (including Nox1, Nox2, Nox3, Nox4, Nox5, Duox1, and Duox2), which was the key pathogenesis of inflammation-dependent carcinogenesis [80]. In addition, HP triggered the up-regulation of four miRNAs (let-7f-5p, let-7i-5p, miR-146b-5p, and −185-5p) that modulate the *CIITA* expression and therefore the HLA−II expression, which resulted in the HP infection persistence and the risk of developing GC in HP-infected patients [81]. To our knowledge, IRSHG was the first molecular prognostic signature specific to HP+ GC patients. IRSHG demonstrated excellent ability in predicting the prognosis of HP+ GC patients, which was better than previously published prognostic signatures for GC [44,45,46,47]. Several immune-related pathways were enriched in the high-risk group, and more intense immune cells infiltration was observed in the high-risk group compared with the low-risk group. In addition, patients with low IRSHG score demonstrated high copy number load and low immune signature score, which was consistent with previous reports [82,83]. Furthermore, the two groups divided by IRSHG showed great difference in mutation profile, pathway enrichment, immunotherapy, and chemotherapy, which might guide the precise treatment of HP+ GC patients.

HP seropositivity was reported to be linked to a detrimental impact on the efficacy of anti-PD-1 immunotherapy in patients [84]. HP eradicating therapy in GC patients could reduce the risk of tumor recurrence and prolong the postoperative survival, while it could not revert the immunotherapy hyporesponsiveness induced by HP infection [84,85,86]. Therefore, a powerful tool was required to personalize the treatment for HP+ GC patients in the context of cancer immunotherapies. To our surprise, IRSHG showed the potential prediction ability for immunotherapy that patients in the low-risk group were more sensitive to anti-PD-1 immunotherapy than those in the high-risk group. The downregulation of MHC I and MHC II was common in many malignant cancers, indicating bad prognosis, distant metastasis, and durable response to anti-PD-1 immunotherapy [87,88,89]. In this study, we noticed that the enrichment scores of MHC I and MHC II calculated by ssGSEA were higher in the low-risk group than in the high-risk group, which might explain the immunotherapeutic sensitivity of anti-PD-1 treatment. For chemotherapeutic drugs, several drugs with potential efficacy were identified for HP+ GC patients in two groups, some of which have shown to be effective in in vitro experiments. For example, staurosporine could reduce the production of granulocyte-macrophage colony-stimulating factor caused by HP infection in gastric epithelia [90]. Several antibiotics (such as norfloxacin [91]) were identified in drug response prediction, and these antibiotics might help the HP eradication therapy and further cancer treatment for HP+ GC patients. In addition, troleandomycin (a kind of antibiotic, CMap score ranked 20/1288) showed great potential in reversing IRSHG-associated gene expression pattern through CMap analysis, which might be suitable for the HP eradication in GC patients with HP infection.

There are two limitations in this study. First, the construction and verification of IRSHG were based on 153 HP+ GC patients, and an external validation cohort of HP+ GC patients should be included to validate the prediction power of IRSHG. Due to the limited sequencing data of HP+ GC patients, we could not find a suitable cohort that was publicly available. With more high-throughput sequencing data becoming available, we believed that IRSHG would further develop and improve. Second, our study was an in vitro study that was carried out based on data mining and bioinformatics analysis, lacking experimental and clinical validation. More convincing evidence and the underlying biological mechanisms of IRSHG might be discovered from comprehensive in vivo and in vitro experiments.

## 5. Conclusions

Overall, we propose a novel prognostic signature named IRSHG, which is the first molecular prognostic signature specially designed for GC patients with HP infection. Comprehensive analyses demonstrate that IRSHG has high reliability and feasibility in predicting the survival outcome of patients with GC. Additionally, IRSHG can also be leveraged to investigate potential therapies, providing a better understanding of personalized treatment of HP+ GC. The efficacy of IRSHG warrants further validation in large prospective cohort studies. Additional in vivo and in vitro experiments are also needed to explore the underlying biological mechanisms of IRSHG in the future. Regardless, we believe that IRSHG holds the potential to become a promising biomarker for prognosis and therapeutic prediction, which may further improve the clinical management of the HP+ GC.

## Data Availability

All data presented in this study was publicly available. The HP infection status of Singapore cohort was provided by Professor Zhengdeng Lei and Professor Steve Rozen.

## Supplementary Materials

The following supporting information can be downloaded at: https://www.mdpi.com/article/10.3390/cancers14133276/s1, Figure S1: Forest plots showing the results of Univariate (A) and Multivariate (B) Cox regression analysis for seven IRGs in IRSHG, Figure S2: ROC curve, Kaplan-Meier survival curve, and Risk score plot for the test set (A) and validation set (B), Figure S3: Calibration curve analysis of the 1-year (A), 3-year (B), and 5-year (C) survival prediction accuracy of the IRSHG-integrated nomogram. Decision curve analysis (DCA) of 1-year (D), 3-year (E), and 5-year (F) overall survival for the clinical utility evaluation of the nomogram, Figure S4: Drug-mutation network displaying association between drugs candidates and corresponding copy number mutations, Figure S5: (A) The working principle of ‘signature reversion’-based computational approach. (B) Correlation between 15902 genes with IRSHG using Pearson correlation analysis. 300 genes with high correlation with IRSHG were selected for CMap analysis. (C) Distribution of CMap scores for 1288 drugs. Top ranked 10 drugs with lowest CMap scores were illustrated, Table S1: Hallmark pathways identified by GO enrichment analysis and KEGG pathway analysis based on IRGs in immune-related modules, Table S2: Hallmark pathways enriched in the high-risk groups based on GSEA, Table S3: Hallmark pathways enriched in the low-risk groups based on GSEA, Table S4: Drug candidates with potential therapeutic effect for the low-risk group or the high-risk group, Table S5: Drug candidates and corresponding biomarkers for HP+ GC patients, Table S6: Correlation between 15902 genes with IRSHG using Pearson correlation analysis, Table S7: The result of CMap analysis.
