# Supplementary material for: An Immune Signature for Risk Stratification and Therapeutic Prediction in Helicobacter pylori-Infected Gastric Cancer
Source: Cancers (Basel). 2022 Jul 4;14(13):3276. doi: 10.3390/cancers14133276 (PMC9265823; doi:10.3390/cancers14133276)
Supplement: Supplementary file 1 [file cancers-14-03276-s001.zip › Supplement figures.pdf]

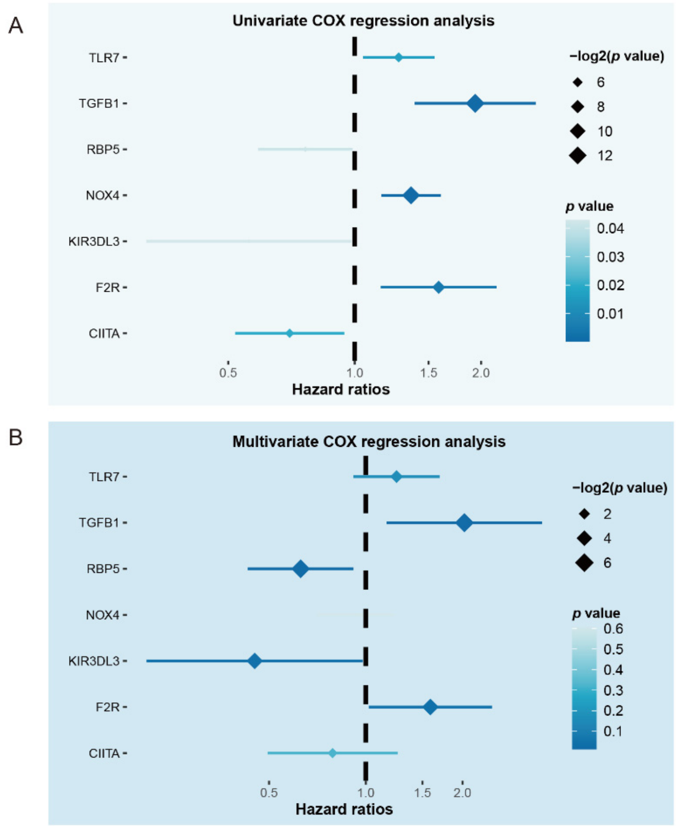

Figure S1. Forest plots showing the results of Univariate (A) and Multivariate (B) Cox regression analysis for seven IRGs in IRSHG.

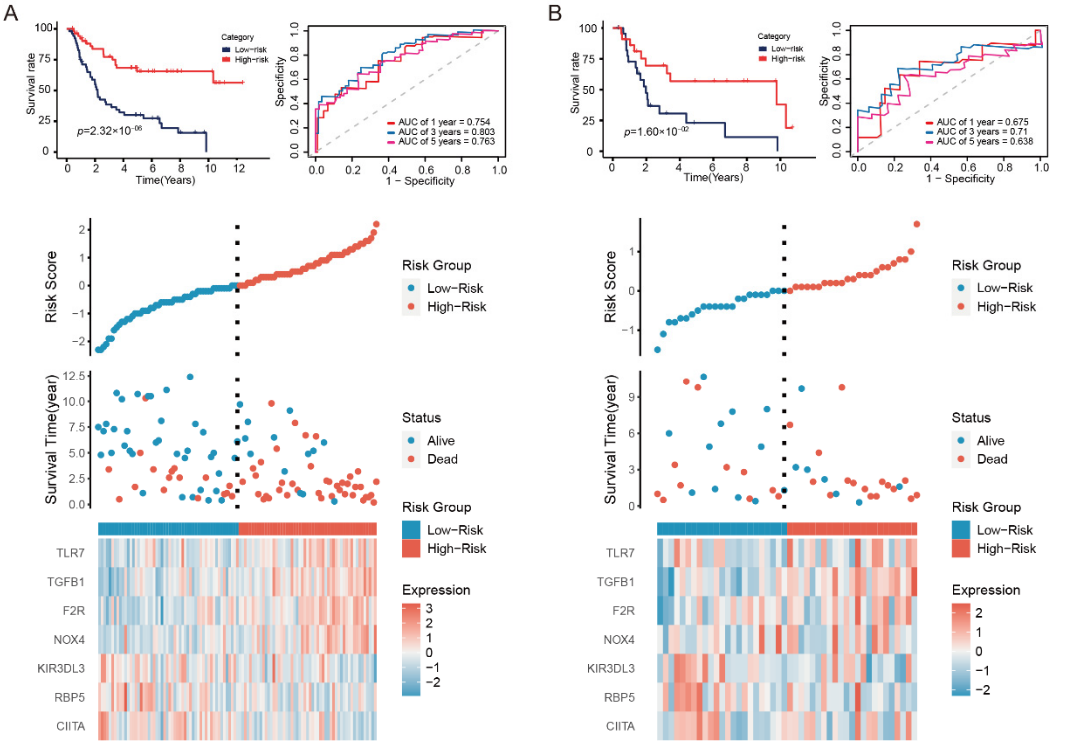

Figure S2. ROC curve, Kaplan-Meier survival curve, and Risk score plot for the test set **(A)** and validation set **(B)**.

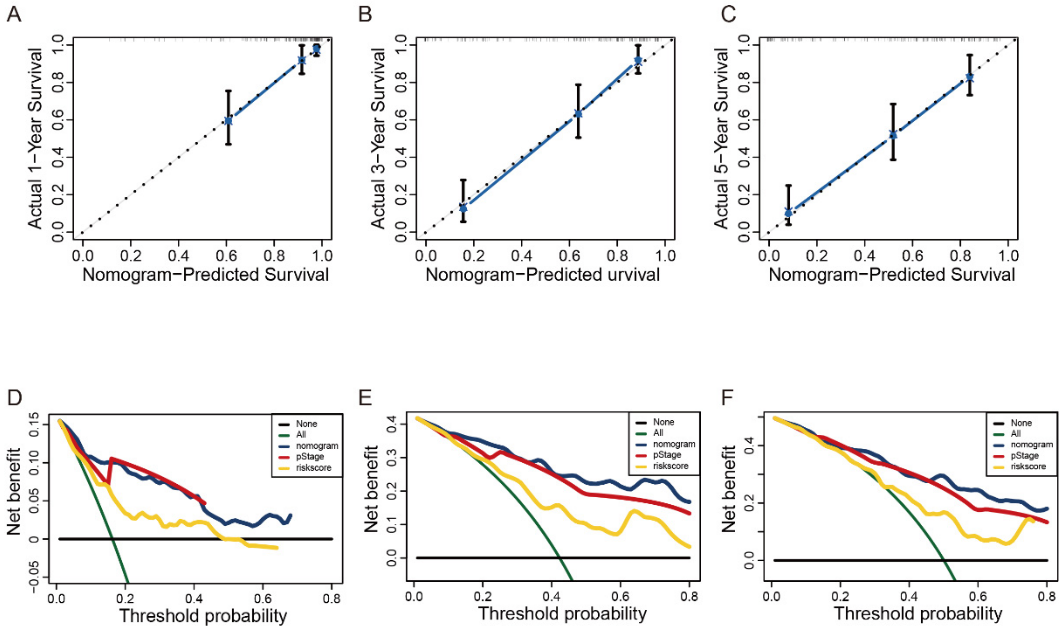

Figure S3. Calibration curve analysis of the 1-year **(A)**, 3-year **(B)**, and 5-year **(C)** survival prediction accuracy of the IRSHG-integrated nomogram. Decision curve analysis (DCA) of 1-year **(D)**, 3-year **(E)**, and 5-year **(F)** overall survival for the clinical utility evaluation of the nomogram.

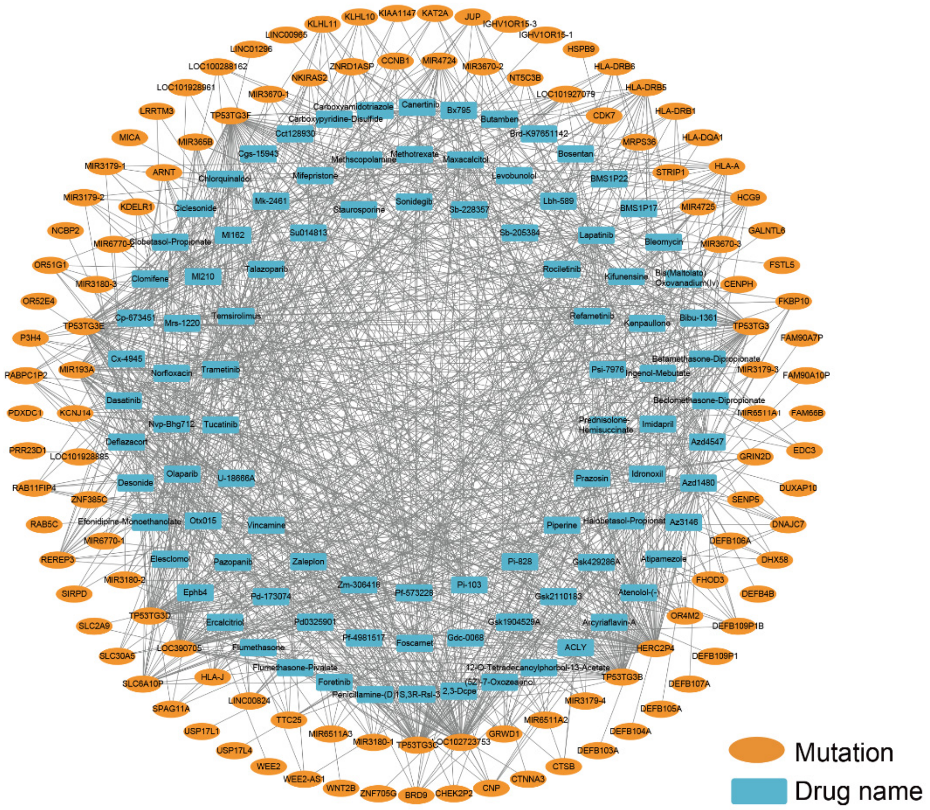

Figure S4. Drug-mutation network displaying association between drugs candidates and corresponding copy number mutations.

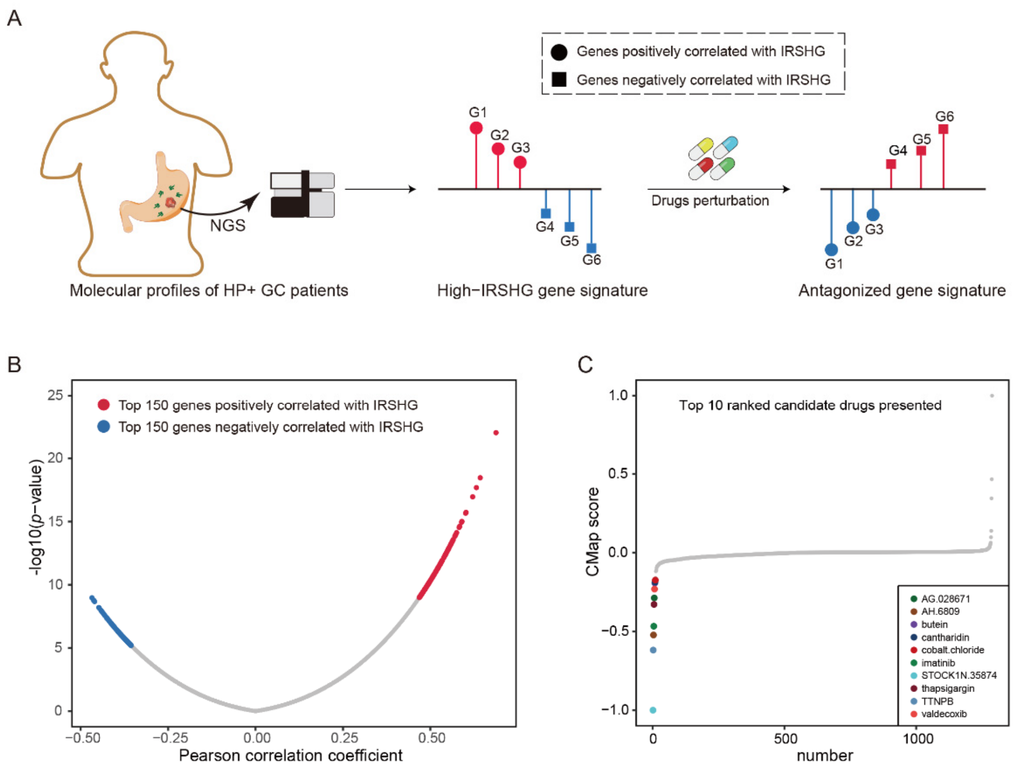

Figure S5. (A) The working principle of 'signature reversion'-based computational approach. (B) Correlation between 15902 genes with IRSHG using Pearson correlation analysis. 300 genes with high correlation with IRSHG were selected for CMap analysis. (C) Distribution of CMap scores for 1288 drugs. Top ranked 10 drugs with lowest CMap scores were illustrated.
